# Isolation and Investigation of Natural Rare Earth Metal Chelating Agents From *Calothrix brevissima* - A Step Towards Unraveling the Mechanisms of Metal Biosorption

**DOI:** 10.3389/fbioe.2022.833122

**Published:** 2022-02-10

**Authors:** Wojciech Jurkowski, Michael Paper, Thomas B. Brück

**Affiliations:** Werner Siemens Chair of Synthetic Biotechnology, Technical University of Munich (TUM), Garching, Germany

**Keywords:** biosorption, cyanobacteria, rare earth elements, mechanism, calothrix, complexation

## Abstract

In this study water soluble compounds that form complexes with Rare Earth Elements (REE) and other metals were isolated from *Calothrix brevissima* biomass with chromatographic methods for the first time. Molecular characterization showed that the isolated compounds are most likely polysaccharides comprised of arabinose, xylose, mannose, galactose and glucose. FT-IR analysis revealed functional groups involved in the binding mechanism of Tb are likely sulfate- and to a lesser extend hydroxyl-groups. The binding specificity of the isolated compounds was investigated with different metal solutions. Here, ions of the alkali and alkaline earth metals Na, K, Mg and Ca showed no competition for Tb-binding even at 10-fold excess concentration. Ions of the elements Co and Pb on the other hand replaced Tb at higher concentrations. Addition of the isolated compounds significantly reduced the precipitation of Eu at pH-values between 6.7 and 9.5, indicating that the interaction between the isolated chelators and Rare Earth Metals is stable even at high pH-values.

## Introduction

**FIGURE 1:**
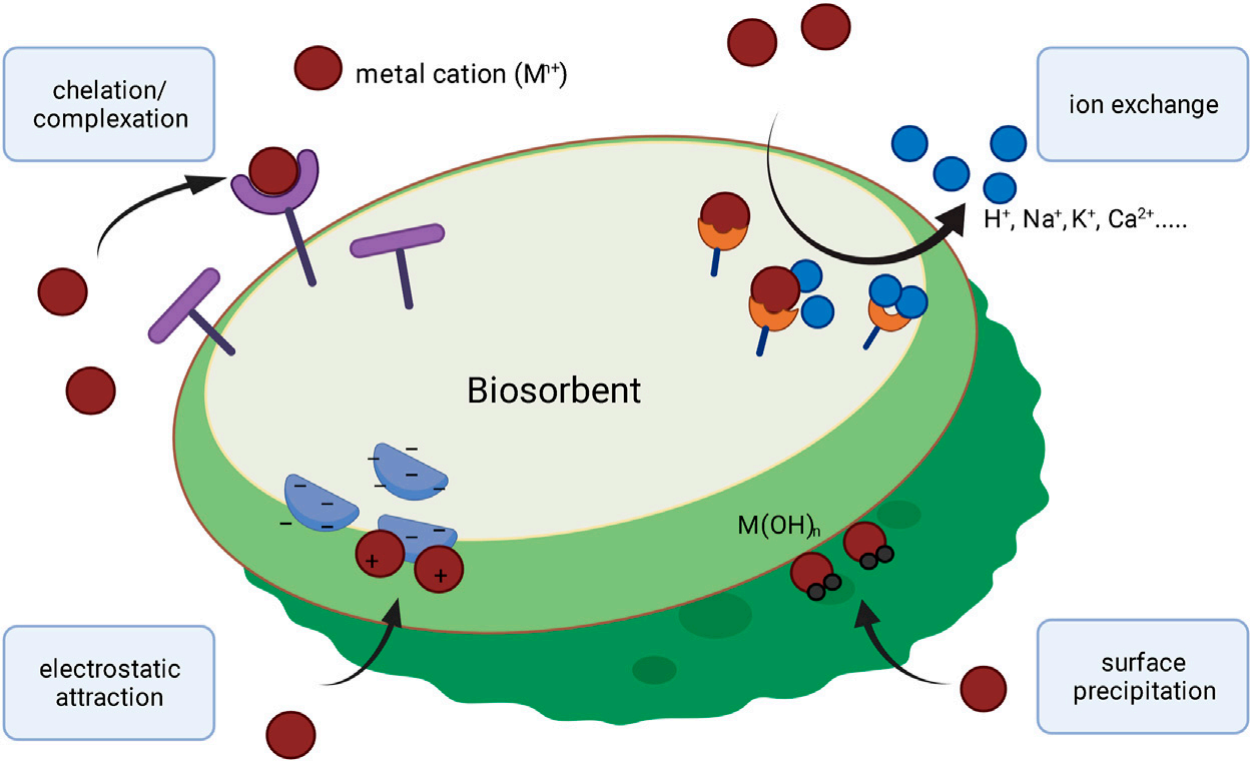
Schematic overview of proposed mechanisms for biosorption of metal-ions.

Many industries, such as metallurgy or mining, discharge enormous quantities of aqueous effluents with relatively high concentrations of heavy metals into the environment. This poses a significant threat to ecosystems and public health. Physical and chemical methods for the removal of metal ions from aqueous effluents have been proposed and applied but these methods are often commercially impractical, either because of high operating costs or the generation of toxic solid wastes (Gavrilescu 2004). A promising environmentally friendly approach for the removal or recovery of metal contaminants from aqueous wastes is biosorption (Abbas et al., 2014). Different living and non-living bio-materials, such as fungal- or bacterial biomass and agricultural waste, have been reported as suitable biosorbents (Kumar et al., 2014; Atiba-Oyewo et al., 2019). So far, various autotrophic microorganisms, including cyanobacteria, green- and brown algae, have also been used and investigated for the removal of metals from aqueous solutions (Romera et al., 2006; Mustapha and Halimoon 2015; Singh 2020). Biosorption of metals is a passive process, that involves the attachment of different elements to the surface of biomass. Various mechanisms have been reported for the binding of metals from aqueous solutions, such as chelation/complexation, surface precipitation or ion exchange (see Figure 1) (Volesky and Holan 1995; Li and Yu 2014; Salam 2019).

Metal biosorption primarily depends on the components of the involved biomass, in particular the cell surface and the spatial structure of the cell wall (González et al., 2011). Several chemical groups on the surface of cells have been proposed to have an influence on the biosorption of metals, such as acetamido, amino, amido, hydroxyl, sulfhydryl, sulfate, phosphate and carboxyl-groups (Gardea-Torresdey et al., 1990; Schiewer and Volesky 2000; Lesmana et al., 2009; Heilmann et al., 2015). Variations in the presence of functional groups on the cell wall surface can be responsible for different biosorption mechanisms which, in turn, can influence the binding affinity or selectivity for certain elements (Gadd 2009). Extracellular polysaccharides (EPS) for instance are known to have a high binding affinity for metals (Liu et al., 2001; Yue et al., 2015). However, other structures like extracellular proteins can also contribute to metal-adsorption (Yang et al., 2020). In this context, metal-accumulation on the cell surface depends on various factors, such as number of functional groups, accessibility of binding sites or the chemical environment (Bilal et al., 2018). The interplay of metal-binding cell wall components and the mechanisms involved in the adsorption process are not yet fully understood. Interest in understanding these underlying mechanisms of biosorption has grown during the past years as the application of biomass can be an effective and sustainable method to recover and recycle valuable metals from industrial waste streams or remove pollutants from waste water (Vieira and Volesky 2000). In order to better understand the mechanisms involved in the adsorption of metals, this study focuses on chemical components in the cell wall of the cyanobacterium *Calothrix brevissima,* that are responsible for the metal-adsorption*. C. brevissima* has already been investigated in the 1950’s as one of the nitrogen fixing phototrophic microorganisms from rice fields of Japan (Watanabe et al., 1951). It belongs to the genus *Calothrix* representing an ubiquitous blue green algae with a complex life cycle and a high variability among the known species. Their characteristic features are the formation of partially branched, filaments of vegetative cells ending in long trichomes as well as a durable sheat (Livingstone and Whitton 1983; Bohuslav 2007). It has been demonstrated on the example of *Calothrix parietina*, that this water insoluble sheath can be extracted and binds more than 0.7% w/w of heavy metal (Weckesser et al., 1979). It is therefore not surprising, that *C. brevissima* was listed as a good biosorbent of lanthanides with a capacity for approximately 0.5 mmol Nd g^−1^ biomass (Heilmann et al., 2015). In this study, we describe the isolation of soluble metal chelating constituents from *C. brevissima* that show interaction with Rare Earth Elements (REE) and other metals. The molecular structure of these compounds and their metal binding mechanism was further investigated by FT-IR spectroscopy and luminescence spectrometric methods. As the adsorption of metals takes place at the cell-surface it can be assumed, that the investigated structures are part of the cell wall. This study provides new insight in the underlying mechanisms for metal biosorption and is significant step towards the molecular understanding of this complex process.

## Materials and Methods

### Cultivation and Harvesting of Biomass

The cyanobacterium *C. brevissima* (SAG Strain Number: 34.79) was maintained in BG-11 medium (Stanier et al., 1971) at 25°C in shaking flasks. Recurring recultivation was done after 4 weeks. The biomass of *C. brevissima* used throughout this experiments was obtained by cultivation in a 2 L glass CSTR photobioreactor (INFORS HT, Switzerland). The gasflow (1 VVM) has been finely dispersed through the sparger mounted below the stirrer turning at 500 rpm. Additional CO_2_ (from 0.5 to 2% v/v adjusted daily) has been supplemented to keep the pH below 7.2 using a gas mixer. The medium used was BG-11 (Stanier et al., 1971), and the temperature was set to 25°C. After a cultivation period of one week, the biomass was harvested by filtration on a porcelain sieve with 3 mm holes. Colonies adhering to the walls of the bioreactor were removed with a spatula. Subsequently the sample was washed with excess of deionized water to remove residues of growth medium, lyophilized and stored in a freezer at -20°C.

### Extraction of Biomass Derived Chelators

**FIGURE 2:**
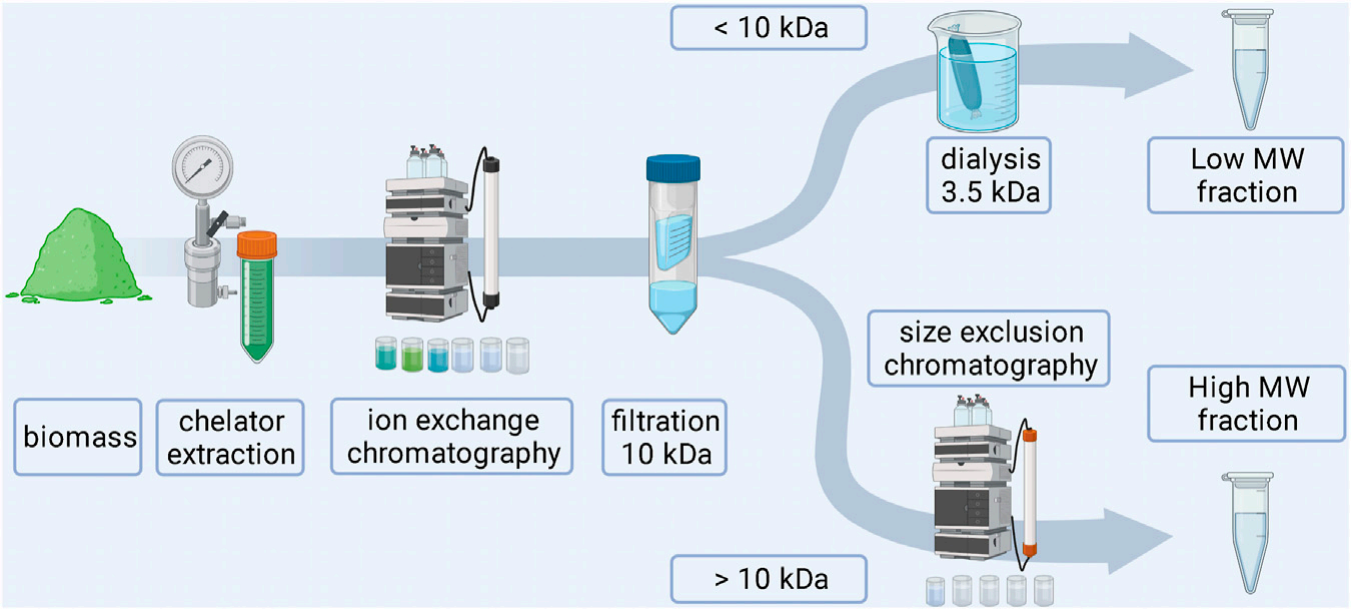
Schematic workflow for the extraction of biomass derived chelators.

The extraction began with cell disruption using a high pressure homogenizer (Avestin Emulsiflex B15, Aventis Europe, Germany) set to 2000 bar. Biomass (250 mg) suspended in 15 ml of deionized water (dH_2_O) was passed through the device three times. The disrupted cells were incubated in a shaker at 50°C for 1 h to dissolve water soluble compounds. The undissolved residue was separated by centrifugation (Eppendorf 5810R centrifuge, Eppendorf, Germany) at 10,000 rcf for 10 min. Prior to the subsequent chromatographic run the supernatant was filtered with a 0.45 µm syringe filter (Filtropur S from Sarstedt Inc., USA) and a 0.2 µm membrane filter in a vacuum vial (Whatman, GE Healthcare, USA). Aliquots of 5 ml extract were injected into the system (NGC Chromatography System, Bio-Rad) consisting of a 5 ml sample loop, two pumps, mixer, UV/Vis detector, conductivity detector and a fraction collector. The separation was performed on a glass column filled with 5 ml anion exchange resin Q-Sepharose fast flow (GE Healthcare, USA). Initially 12 ml of dH_2_O were pumped in order to pass the sample through the column and allow negatively charged molecules to bind to the resin. Subsequently a gradient of NaCl (0–1 M) was run for 15 min and 1 M NaCl was run for another 15 min to ensure full regeneration of the resin. The flow rate of 0.6 ml min^−1^ was constant for all steps. The collected fractions of 2.5 ml were probed for Tb luminescence sensitization according to a previously published protocol (Jurkowski et al., 2020). Further purification of the extracts was initiated by using a 10 kDa centrifugal filter concentrator (Centriprep, Merck, Germany). Both fractions were kept for further analysis. The fraction that was below the cut off limit of the filter was desalted using a dialysis membrane with a molecular weight cut off at 3.5 kDa (Spectra/Pro, Spectrum Laboratories Inc., USA) against H_2_O until a conductivity of 0.01 mS cm^−1^ was obtained. The fraction that was retained by the 10 kDa filter was further purified using size exclusion chromatography with the same chromatographic system as before. For this purpose, a Tricorn Superdex 75 (GE Healthcare, USA) column was used. The flow rate was set at 0.5 ml min^−1^. Fractions of 2.5 ml were collected and metal binding molecules were detected with the Tb sensitization method reported previously (Jurkowski et al., 2020). Thereafter, all samples were dried with a vacuum concentrator (HT-4 Atlas Evaporator) linked to a VC 3000 Vapour Condensator (both from GeneVac, United Kingdom) for storage and further analysis. A schematic overview for the isolation of biomass derived chelators is shown in Figure 2.

### Molecular Characterization

**FIGURE 3:**
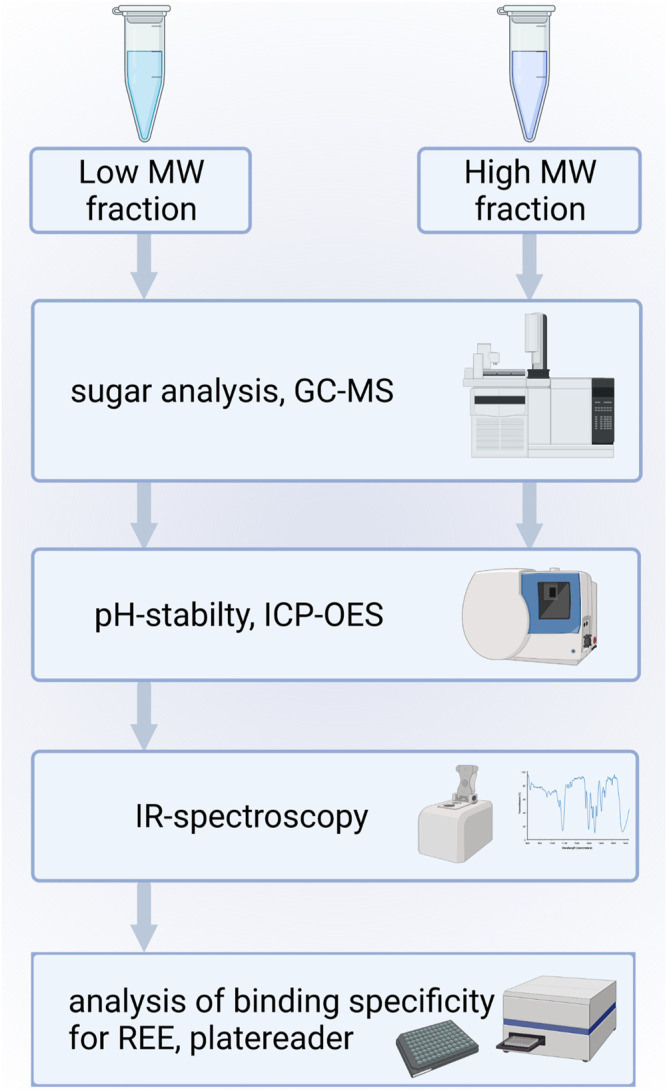
Schematic workflow for the molecular characterization of isolated metal chelators.

The isolated biomass derived metal chelators were further characterized by analyzing their chemical composition and mode of interaction with metals. A schematic overview for the isolation of biomass derived chelators is shown in Figure 3.

### Sugar Analysis

As cell wall components in cyanobacteria are composed of chemically complex polymeric carbohydrates, the monomeric sugar composition of extracted Tb chelator samples were additionally analyzed with a GC-MS based method. For the analysis of sugar monomers each Tb interacting sample has been hydrolyzed with 1% H_2_SO_4_ in an autoclave for 1 h at 121°C at 1 bar in order to release monomeric carbohydrate building blocks from the sample constituting polymeric carbohydrates. Each sample was then centrifuged for 10 min at 10,000 rcf. Following hydrolysis, the solution has been neutralized with calcium carbonate to a pH of 7. Precipitated calcium salt has been removed by centrifugation (30 min, 10,000 rcf) immediately after the neutralization. The supernatant was frozen at −40 °C for 48 h. After the sample was heated to 5°C and then the sample was centrifuged (30 min, 10,000 rcf) to remove any residual precipitate. The first step after centrifugation was to obtain an estimate of possible carbohydrate building blocks that constitute each sample to facilitate the GC-MS evaluation. This was achieved by using a HPLC Agilent Infinity II LC 1260 system (Agilent technologies, Waldbronn, Germany), equipped with an autosampler, quaternary pump, column oven, DAD and a Shodex RI detector (Showa Denko Europe GmbH, Munich, Germany). Prior to injection each sample was filtered with Modified PES 500 µL Centrifugal Filters (VWR, Ismaning, Germany) with a cut-off of 10 kDa. In a subsequent step, the monomeric sugar mixture resulting from chemical hydrolysis was analyzed using *via* HPLC. A Rezex ROA-Organic Acid H+ (8%) ion-exclusion column (300 mm, 7.8 mm internal diameter; Phenomenex LTD, Aschaffenburg, Germany) was used for the isocratic separation with 5 mM sulfuric acid at a flow rate of 0.5 ml/min at 70°C. After HPLC data evaluation, the remaining sample material was processed with a trimethylsilyl (TMS) derivatization for subsequent GC-MS based identification of the carbohydrate constituents. For this, a modified version of a previously described protocol was used (Gorner et al., 2016). 50 µL of pyridine were added to each sample. Thereafter, 50 µL of MSTFA (N-Methyl-N-(trimethylsilyl)trifluoroacetamide) with 1% TCMS (Trimethylchlorosilane) was added and the samples were incubated in a water bath at 50°C for 1 h. GC-MS analysis was carried out using a modified version of a previously described protocol (Ringel et al., 2020). The samples were analyzed using a Trace GC-MS Ultra system with DSQII (Thermo Scientific, USA). One microliter (1/10 split ratio) of the respective sample was injected by a TriPlus auto sampler onto a SGE BPX5 column (30 m, I.D. 0.25 mm, film 0.25 µm) with an injector temperature of 280°C. Helium gas was used as carrier with a flow rate of 0.8 ml min^−1^. The initial oven temperature was set to 70°C for 2 min. The temperature was subsequently ramped to 290°C with a rate of 5°C min^−1^ and then held for 4 min. MS data was recorded at 70 eV (EI). Masses were recorded in positive mode in a range between 50 and 650 m/z.

### pH-Stability of Chelates

In preparation of these measurements various lanthanide metals (Ce^3+^, Tb^3+^, Eu^3+^) were incubated with the biomass derived chelator samples in order to identify the metal-chelator interaction, which provided the best spectrophotometric signal to noise ratio. In that regard the interaction of all biomass derived chelators with Eu^3+^ was superior to all other combinations. To evaluate the pH stability of the resulting biomass derived lanthanide chelates, Eu^3+^-solutions were mixed with buffer solutions at pH values between 5.9 and 9.5.5 µL of a 10 mM europium(III)nitrate-solution were added to 50 µL of each fraction. The samples were filled up to 1 ml with 10 mM PIPES (1,4-piperazinediethanesulfonic acid) buffer set to pH-values of 5.9, 6.7, 7.5 and 8.0 as well as a 10 mM CAPS (N-cyclohexyl-3-aminopropanesulfonic acid) buffer at pH 9.5. To remove precipitated europium, all samples were centrifuged for 20 min at 10,000 rcf. For the determination of the remaining europium concentration 500 µL of supernatant were used for measurement *via* ICP-OES (Inductively Coupled Plasma Optical Emission Spectrometry) (Agilent 725 Series ICP Optical Emission Spectrometer, Agilent Technologies Inc., USA).

### IR-Spectroscopy

Attenuated total reflection infra-red (ATIR) spectra were obtained from each dry sample to determine the functional groups in the absence of rare earth metal binding (Coates 2000). In a similar experiment, samples were incubated with 1 µmol terbium(III)nitrate and again measured *via* ATIR to identify changes in the spectral properties of respective functional groups. Specifically, samples were mixed with the metal solution by iterative pipetting steps and subsequently dried in the vacuum concentrator. Finally, spectra were recorded with an FT-IR spectrometer equipped with a diamond ZnSe UATR (Universal Attenuated Total Reflectance) polarization accessory (Frontier, PerkinElmer, USA).

### Binding Capacity

In order to determine the maximum binding capacity on the example of Tb, a derivation of the previously developed luminescence excitation method (Jurkowski et al., 2020) was applied. When using the biomass specific excitation wavelength, unbound Tb-ions exhibits negligible luminescence at sub-millimolar concentrations in comparison to Tb chelates with the sample. Therefore, any increase of measured Tb emission intensity can only be attributed to more Tb-ions bound to the biomass (concentration dependent quenching can be excluded in highly diluted solutions). 50 µL of the isolated REE-interacting fraction at a gravimetrically determined concentration of 4.0 mg/ml were transferred into a quartz glass 96 multiwell plate (Hellma Analytics, Müllheim, Germany) and spiked with 5–20 µL of a 10 mM terbium(III) nitrate solution. The sensitized luminescence of Tb was measured for every sample at an excitation wavelength of 230 nm and an emission wavelength of 544 nm using a EnSpire multiplate reader (PerkinElmer, USA).

### Binding Specificity

50 µL of each fraction was subsequently spiked with 6 µL of a terbium(III)nitrate solution with a concentration of 10 mM to reach a final concentration of 0.2 mM, giving a saturated sample. Different metal-solutions were added for final concentrations of 0.1, 0.2, 0.7 and 2.0 mM. The pH-value of all metal-solutions was prior set to 5 ± 0.2. Each well was filled to a final volume of 300 µL with demineralized water. As potential metal compounds that could interfere with the binding of terbium, sodium (NaCl), magnesium (MgCl_2_), potassium (KCl), calcium (CaCl_2_), cobalt (Co(NO_3_)_2_), lead (Pb(NO_3_)_2_) and lanthanum (La(NO_3_)_3_) were tested. Metal salts were bought from Carl Roth GmbH & Co. KG, Karlsruhe, with a purity ≥99%. All samples were transferred into a quartz glass 96 multiwell plate (Hellma Analytics, Müllheim, Germany) and excited with a wavelength of 230 nm and the emission spectrum from 460 to 570 nm was measured using an EnSpire multiplate reader (PerkinElmer, USA). Displacement of Tb-ions by other metal ions was observed by comparing the emission signal intensities at 544 nm.

## Results and Discussion

### Cultivation and Extraction Method

**FIGURE 4:**
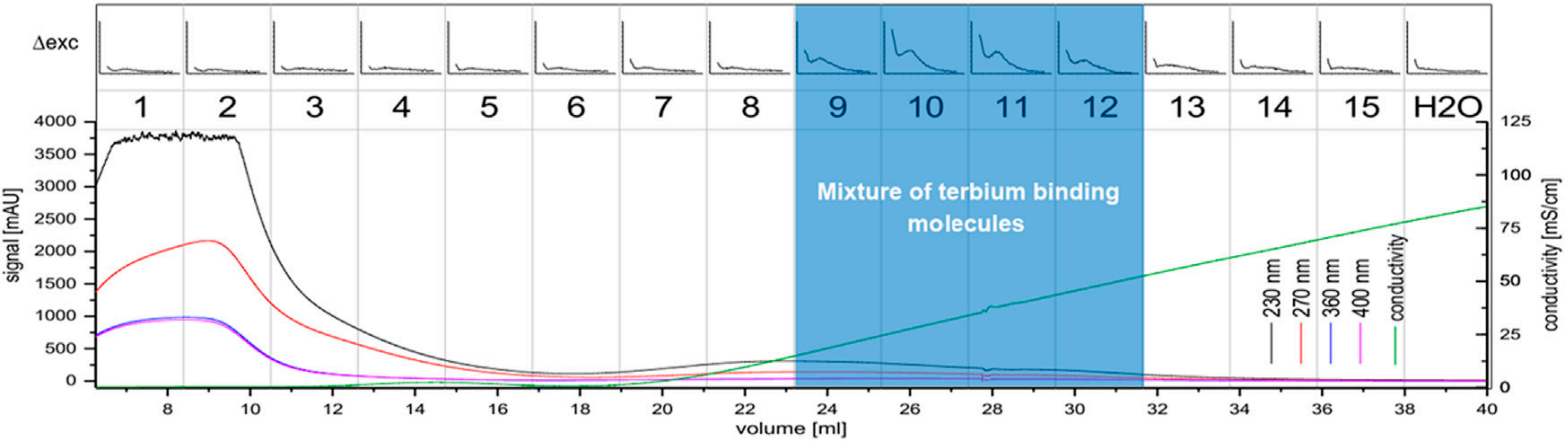
Anion exchange chromatography of the *C. brevissima* biomass extract; The UV-signal in mAU is plotted against the elution volume from the wavelengths 230 nm (black), 270 nm (red), 360 nm (blue) and 400 nm (pink). The conductivity is displayed in green. 2.5 ml fractions were collected (numbered 1-15). Each fraction was tested for interaction with Tb *via* luminescence spectrometry. The ∆RFU-values (before and after addition of Tb) measured at 545 nm (200–5000, if not noted otherwise) are plotted against the excitation wavelength (230–402 nm). Also, the H_2_O-background measurement is displayed. Fractions 9-12 were pooled.

**FIGURE 5:**
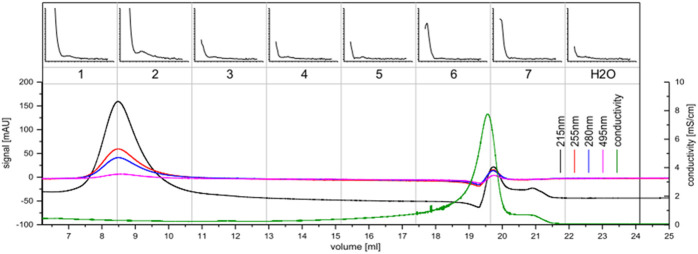
Size exclusion chromatography of the concentrate of *C. brevissima*; The UV-signal in mAU is plotted against the elution volume for the wavelengths 215 nm (black), 255 nm (red), 280 nm (blue) and 495 nm (pink). The conductivity is displayed in green. 2.5 ml fractions were collected (numbered 1-7). Each fraction was measured via luminescence spectrometry. The ∆RFU-values (before and after addition of Tb) measured at 545 nm (200–5000, if not noted otherwise) are plotted against the excitation wavelength (230–402 nm). Also, the H_2_O-background measurement is displayed. Fractions one and two were pooled for further analysis.

During cultivation, *C. brevissima* has formed macroscopic colonies encapsulated in a remarkably stable biofilm, which adhered to the glass walls of the reactor. This fact has simplified harvesting as well as dewatering thus reducing the cost of necessary equipment and energy which makes it a suitable industrial strain. Dried samples were mixed with water and homogenized under high pressure. The lysed cells resulted in a faint yellowish coloration of the supernatant. Insoluble fractions remained as a pellet after centrifugation. After the removal of supernatant, the pellets have been resuspended in deionized water and incubated in a shaker to allow the weakly soluble compounds to dissolve. While this procedure has been repeated three times, each supernatant was tested for its potential to sensitize Tb luminescence. There was no difference between the first and second wash of the pellet, while the third and fourth repeat yielded decreasing amounts of active molecules. It can be therefore concluded, that about 60 ml of water are necessary to dissolve most of the chelators contained in 250 mg of biomass. The use of buffers has been investigated by performing the procedure with sodium acetate (0.1 M) and TRIS-HCl (0.1 M), both at pH seven instead of deionized water. No difference in solubility could be detected. Therefore, the final protocol included deionized water only. The chelators could be separated from the bulk of other soluble compounds using anion exchange chromatography and were present in fractions 9 to 12 (see Figure 4), which were pooled for further processing. Already this step has yielded a good purity judging by the low UV absorption as compared to the first three fractions which did not interact with Q-Sepharose and were eluted immediately. After the centrifugal ultrafiltration significant precipitation has been observed in the fraction containing lanthanide chelator compounds indicating, that saturation has been reached as a consequence of volume reduction. By transferring this sample to a larger volume of deionized water most of the molecules could be resolubilized. Nevertheless, centrifugation prior to size exclusion chromatography produced a small pellet containing the metal binding molecules still attached to bulkier, insoluble cell fragments. Ensuring that only soluble molecules were injected was not only a measure against column clogging, but more importantly a route to exclude carry-over of non-metal binding compounds prior to characterization. As shown in Figure 5 a high and a low molecular weight fraction that showed interaction with Tb could be separated. The purified sample (fraction 1-2) shows a much higher sensitization effect when compared to fractions collected after anion exchange. Due to the harsh purification conditions it is feasible, that the compounds with a lower molecular weight (faction 6-7) consist of severed building blocks of the larger compound.

### Molecular Characterization

#### Sugar Analysis

**FIGURE 6:**
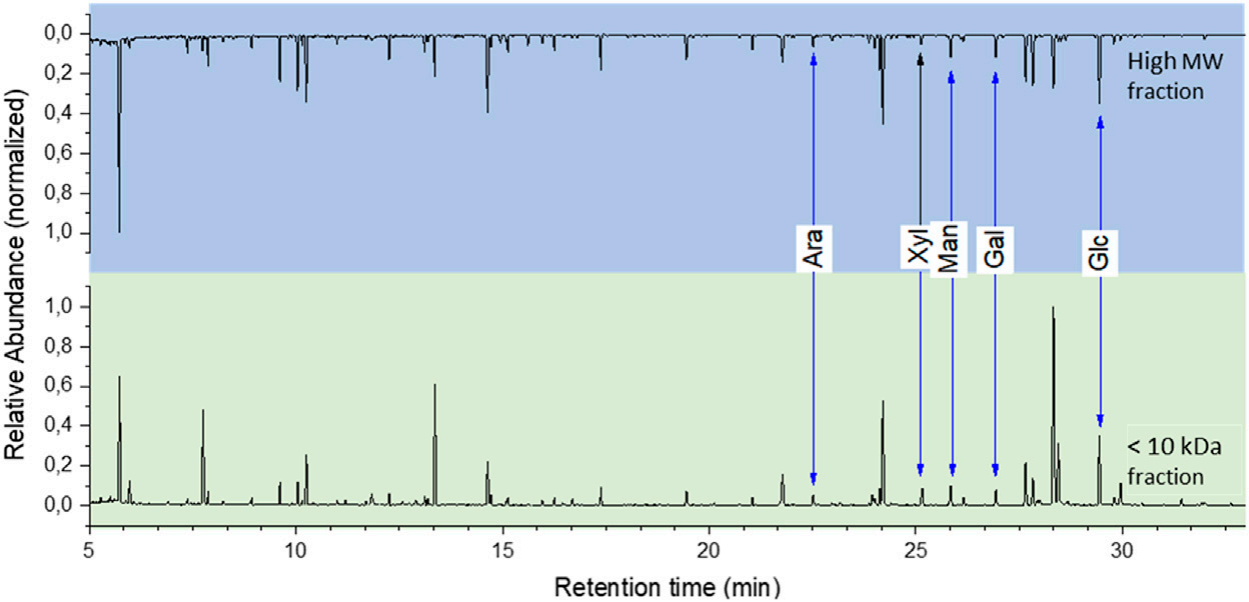
GC-MS sugar analysis of the isolated fractions.

A chemical hydrolysis with 1% H_2_SO_4_ and GC-MS analysis confirmed that both compounds have a similar chemical composition. The evaluation of GC-MS data showed that both the high molecular weight and <10 kDa fraction of the biomass derived lanthanide chelators are composed of arabinose, xylose, mannose, galactose and glucose respectively (see Figure 6). Rhamnose and fructose were not detected. This further supports the assumption that the chelators in the <10 kDa fraction originate from the larger isolated compound.

#### pH-Stability of Chelates

**FIGURE 7:**
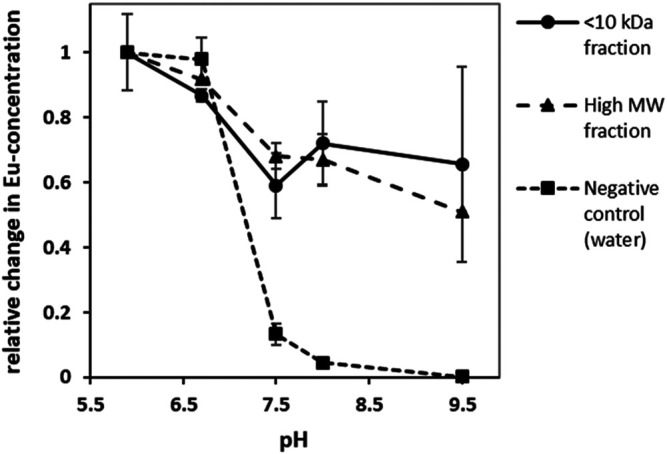
Change in europium-concentration at different pH-values; without the addition of REE-interacting chelators europium precipitated at high pH values leading to a decrease in Eu-concentration. In presence of REE-interacting chelators however a significant proportion of europium remained in solution. (error bars indicate ±*σ*, *n* = 3).

Most heavy metal ions, including lanthanides form insoluble hydroxides and carbonates, which precipitate from the solution when the pH rises above a certain threshold (Plancque et al., 2003). They can be however kept in the solution when a stable chelate is formed. Thus measuring the chelate dissociation as a function of rising pH provides an empirical guideline for the applicable pH range of the chelator. In our experimental setup Eu^3+^ was chosen as a model lanthanide due to the lowest detection limit in optical emission spectroscopy ensuring highest possible resolution. The Eu-concentration was expected to drop significantly above a pH of 6.7. This was confirmed in a negative-control test in which Eu^3+^, solubilized in purified water, was mixed with the different buffer solutions. Here, about 95% (w/v) of the originally dissolved metal ions have precipitated at a pH between 7.5 and 8.0. In contrast to this observation, over 60% (w/v) of Eu^3+^ -ions remained in solution up to a pH of 9.5 (see Figure 7) when the isolated fractions were added. This indicates that the chelates are stable even at high pH values. Moreover, the applied Eu^3+^ concentration was above the determined binding capacity of approx. 0.13–0.25 μmol mg^−1^ for Tb for both fractions and free metal ions were present in the solution. Those would precipitate as quickly as in the negative control between pH of 6.5 and 7.5 thus explaining the steep fall of the curve in this region.

#### FT-IR Spectroscopy

**FIGURE 8:**
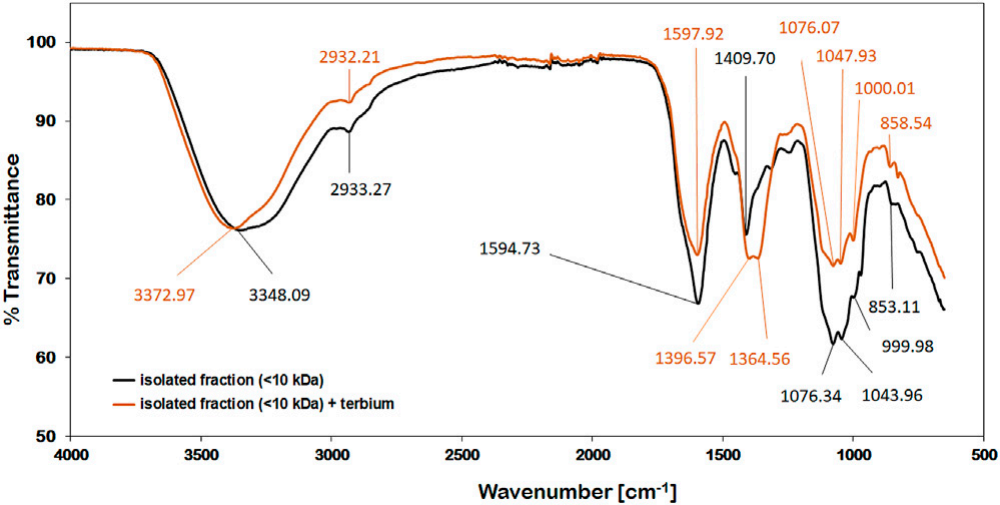
IR-spectra of the isolated (<10 kDa) compounds; before (black) and after adding 1 µmol of Tb^3+^ (orange).

FT-IR spectroscopy is a useful tool for the qualitative measurement of organic functional groups. In this study, IR spectroscopy was used to identify and characterize functional groups in the isolated <10 kDa fraction that interact with Tb. The IR-spectra obtained from this fraction showed many similarities with other polysaccharide IR-spectra (Qian et al., 2009). The strong broad band in the region of 3348 and 3373 cm^−1^ in the IR-spectrum can be assigned to the stretching vibrations of hydroxyl groups (Qian et al., 2018), whereas the absorption bands at about 2932 and 2933 cm^−1^ can be related to the C-H stretching vibrations of CH_2_ groups (Bhattacharya et al., 2014). The signals at 1595 and 1598 cm^−1^ could be antisymmetric stretching peaks of carboxylate ions (He 2015). Literature data (Kacurakova et al., 2000) suggests, that the signals around 1076 and 1047 cm^−1^ as well as a characteristic peak close to 1000 cm^−1^ may be assigned to ß-linked arabinans and ß-galactans respectively. The absorbance at 859 and 853 cm^−1^ could be caused by the presence of *α*-type glycosidic linkages (Ruperez and Leal 1987). As can be seen in Figure 8, the most pronounced change of the spectrum upon metal binding appeared at 1409 and 1365 cm^−1^. This shift could be assigned to asymmetric stretching vibrations of organosulfate groups (Coates 2000). The coordination of these sulfate groups with Tb^3+^-ions might change their IR-signals towards resembling sulfonates which can typically be found between 1365 and 1340 cm^−1^ (Coates 2000). Sulfated sugars in polysaccharide chains that are part of the cell wall have been reported for various cyanobacteria in the past (Hayashi et al., 1996; Ngatu et al., 2012; El-Baky et al., 2014). Besides this indication for Tb-interaction, the weak signal shift of the hydroxyl stretching vibration from at 3348 cm^−1^ to 3373 cm^−1^ could indicate that these functional groups are also involved in the binding mechanism to a small extent.

#### Binding Capacity and Specificity

**FIGURE 9:**
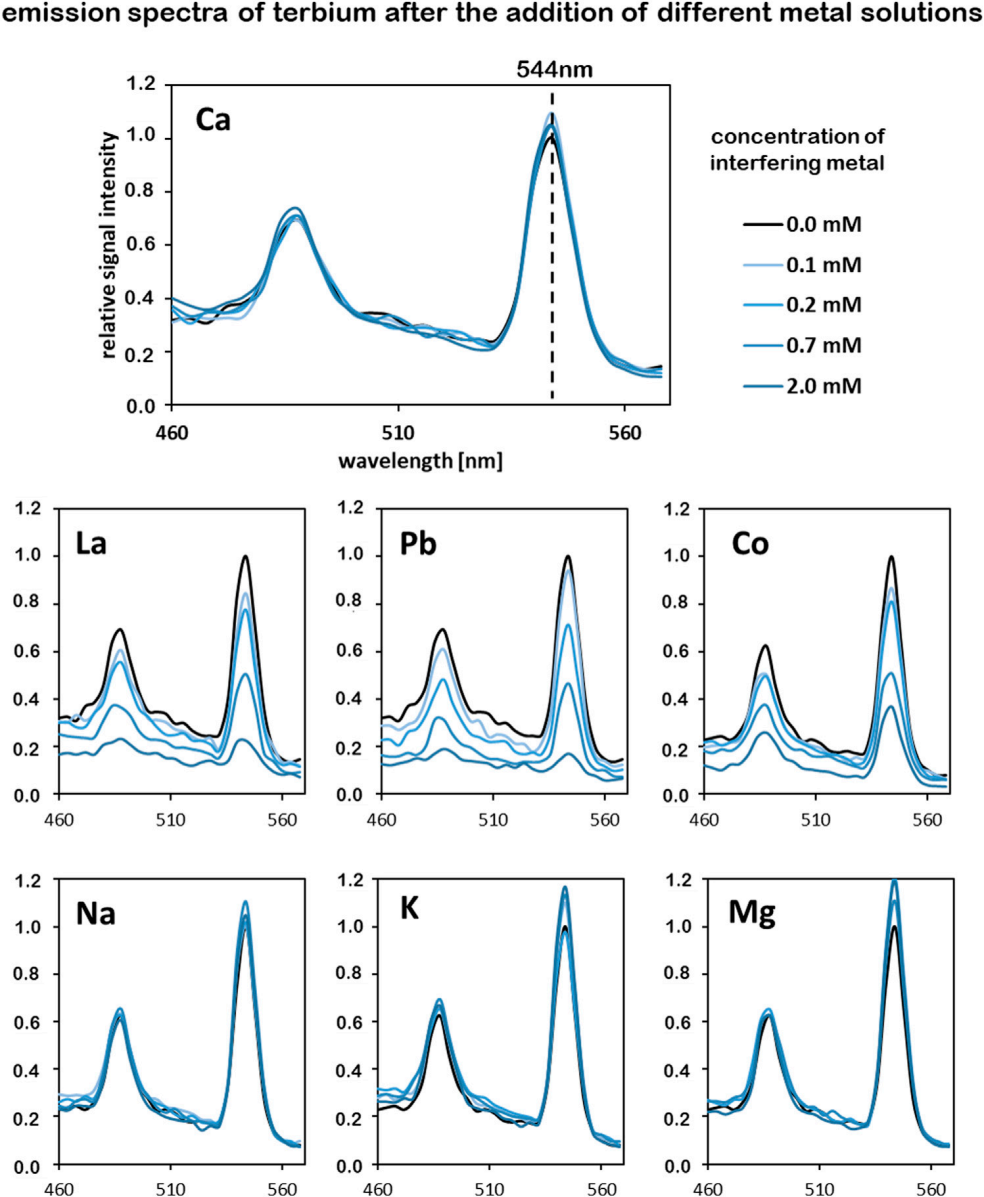
Competition of different metals for complex formation with the isolated chelators (<10 kDa), tracked by analyzing the emission intensity of terbium; Tb concentration in all samples is 0.2 mM. Interfering metals were added in concentrations between 0.1 and 2.0 mM, excitation wavelength was set to 230 nm.

The binding of terbium of the isolated chelators was analyzed *via* luminescence spectroscopy. Due to their weak molar absorptivity lanthanides have low luminescence quantum yield. However, the quantum yields can be highly enhanced by chelating with suitable organic ligands that transfer energy intramolecularly to the emitting lanthanide ion (sensitization) (Al-Kindy et al., 2019) or provide shielding from solvent-induced non-radiative relaxation (Bunzli and Piguet 2005). The isolated chelators from *C. brevissima* biomass form a complex with Tb^3+^-ions, which is more sensitive to excitation at a wavelength of 230 nm resulting in a higher emission intensity. Typically, Tb exhibits a strong emission signal at a wavelength of ca. 544 nm (Li and Korshin 2002). The replacement of Tb-ions by other metal ions results in a decreased signal intensity at this wavelength because of the reduced amount of luminescent Tb-complexes. In our experimental setup with a low Tb-concentration of 0.2 mM only the sensitized ions emit a detectable signal. Consecutive addition of Tb to the isolated metal chelators leads to an increased signal intensity until all available binding sites are saturated. Thus, the binding capacity for Tb of the compounds in the <10 kDa fraction could be roughly estimated to be between 20–40 mg/g. In order to analyze the binding specificity for terbium different metal solutions were added to samples in which all binding sites were saturated with Tb. A decrease in signal intensity at 544 nm indicates a replacement of Tb in the formed complexed by these added metals. Figure 9 shows changes emission spectra of 0.2 mM Tb-solutions after the addition of different metal solutions with concentrations between 0.1 and 2.0 mM. In the conducted experiments the addition of alkali and alkaline earth metals (Na, K, Mg, Ca) did not result in a decreased signal intensity at 544 nm, indicating that these elements do not interfere with complex formation. Increasing the concentration of Co, La and Pb however reduced the intensity of the characteristic emission band for Tb. These elements seem to compete with Tb during complex formation. A *t*-test analysis comparing the changes in emission intensity at 544 nm indicated a significant difference (*p* < 0.05) between the non-interfering metals (Na, K, Mg, Ca) and the metals Co, La and Pb at all tested concentrations between 0.1 and 2.0 mM.

## Conclusion and Outlook

Biosorption is a promissing eco-friendly method for wastewater treatment and metal recovery. Yet, most research focused on the laboratory scale. Low mechanical resistance and stability of biosorbents remain challenging for industrial applications. Nevertheless, biosorption has many advantages, for instance, easy recuperation of adsorbed material, high efficiency at low metal concentrations and cost efficient biomass production (González et al., 2017). Fungal and bacterial biomass as well as agricultural waste have been investigated as promising biosorbent materials (Abbas et al., 2014; Atiba-Oyewo et al., 2019; Gallardo et al., 2020). Biomass derived from algae and cyanobacteria is of special interest for the development of biosorbtion materials because of the presence of many functional cellular compounds that promote metal adsorption, which lead to high adsorption capacities (Kanchana et al., 2014). In this study, negatively charged polysaccharide-based cell wall components derived from biomass of the cyanobacterium *C. brevissima* were sequentially purified *via* chromatographic methods and subsequently characterized by differential spectroscopic methods. Interestingly, these cell wall polysaccharides showed a strong interaction with lanthanides particularly with Tb. These saccharide-based compounds were separated in a high molecular weight and a low molecular weight fraction. The former presumably is a subunit of the larger compound as analysis indicated that both the small and the large components are made up of identical monomeric sugars. Molecular characterization of the small subunit showed the metal-chelating compounds are sulfated polysaccharides. These sulfate groups could be the major contributing factor in the complexation of metal-ions. Interaction between the isolated polysaccharides and REE was demonstrated to be present over a broad neutral to alkaline pH-range (pH 6-9). Interestingly, competitive binding experiments revealed that alkali and alkaline earth metals do not compete with REE in formed complexes, whereas the metals Co and Pb can replace them at high concentrations. Previous studies by Crist et al. suggested that Ca or Mg ions can be replaced by other elements during biosorption as part of an ion-exchange mechanism (Crist et al., 1992; Crist et al., 1994). This was confirmed in a study by Sulaymon et al., in 2013 (Sulaymon et al., 2013). In this context, the binding of competing metals can depend on the functional groups of the biosorbent and ionic properties of the metals, such as electronegativity, ionic radius or redox potential (Naja et al., 2009). Further studies are required to fully understand this phenomenon, as in our experiments neither metal ion charge nor radius were decisive factors for REE binding selectivity to the functionalized polymeric sugar constituents of the *C. brevissima* cell wall. Further, fast kinetic studies should be carried out to investigate the mechanism of competitive binding in more detail.

## Data Availability

The raw data supporting the conclusion of this article will be made available by the authors, without undue reservation.
